# Co-Operative House Buying

**Published:** 1898-05-07

**Authors:** 


### MODERN SOCIOLOGY.

### CO-OPERATIVE HOUSE BUYING.

EVERYTHING is formed into a limited liability company now. The food we eat, the water we drink, the raiment we put on, the furniture of our house, the gas or electric light we burn therein, are all provided by the Somebody Company (Limited), and why not the house itself? Why not? echoes the "Co-operative Land and House Purchase Provident Society (Limited)," the prospectus of which lies before us. Whether it is wise to own the house one occupies is, of course, a question for each man to answer for himself. For a young man, who is not sure that he is in the place where he will spend the rest of his life, it may be better to pay rent for a few years, rather than to buy a house which, when he desires to shift his camp, he may not be able to dispose of to advantage. But as soon as we feel settled for life, when we see the limit to which it is wise to curb our ambitions, the radius within which our daily walk will lie for the remainder of our earthly career, we are conscious of the longing for a home of our own, a place from which we cannot be ejected, and which we may arrange to suit our own taste, as we cannot always do in a landlord's house. But to save up money to buy a house while paying rent all the while; there's the rub! The income of many, one might say of most people, will not permit of that. One can, it is true, raise money on mortgage on the security of the house one buys; but, in the first place, no mortgagee will advance the whole purchase-price of a house; and it may be as difficult to save the first £100 as any part of the rest; and, in the second, the interest on the mortgage is simply a form of rent (though perhaps a smaller one than the ordinary landlord would be contented with), and when you have paid interest for twenty years you are as far from really owning your house as ever. You still owe the amount of your mortgage.

Ordinary building societies also require that a considerable part of the purchase-money should be paid down at once, or alternatively, that the would-be house owner should have been a subscriber for years before he achieves his desire. The co-operative society we have spoken of attempts to do away with this delay, while taking every reasonable precaution against loss. The plan adopted is similar to that of the Massachusetts co-operative banks, which have been a great success; and the English company seems to be formed on lines which are more favourable to the purchaser than the American ones. The idea is to bring into the society a number of outsiders, preference shareholders, interest on whose shares at the rate of 5 per cent. per annum is guaranteed by the property the company holds. These shares, of £1 each, form the first working capital. Ordinary shares of £12 10s. each are to be taken out by those who wish to buy houses through the agency of the company. These shares are paid up at the rate of 1s. 3d. per week, and each share entitles the shareholder to hold land or property to the value of £50. According to the kind of house a man wants to buy he must increase the number of his shares. Thus, for a house or land of the value of £150, a man must subscribe for three ordinary shares; for property to the amount of £350 his shares must number eight, and so on, up to property amounting to £800 in value. Shareholders have, however, no liability beyond the amount of their shares, and the security, land or houses of freehold or long leasehold tenure, is sufficiently good.

It has been decided, in view of the fact that the applicants for ordinary shares are very numerous, that members must be of twelve months' standing before they can apply to the society to purchase any property for them. They can choose their own site and locality, but it must be approved of by the company's surveyor, and for this survey a fee of 10s. 6d. or a guinea, according to the size of house desired, is charged. When the plans are passed the house is begun at once, and can be occupied by the shareholder as soon as it is ready. He is still a tenant; but the rent he pays to the society in the form of paying up his shares is credited to him, and as soon as his shares are matured and they, with the profits accrued to them, amount to the cost of his house, the title deeds of the house are handed over to him. He has become his own landlord. The society hopes that the profits on the shares may, through judicious management, amount to 25 per cent. on the rent paid by the members. This is a great deal to hope for, and most members will probably be glad of profits which are much more modest in amount. These shares, though they cannot be withdrawn, may be transferred and bequeathed.

If the would-be householder prefers it he can gain his object less immediately by becoming a subscriber for the society's bonds. These bonds may be taken out to run any specified period of years, and the subscriptions are payable in monthly instalments of from 1s. 3d. to 10s. per week, as the bondholder may decide when taking out the bond. These bonds bear interest at the rate of 3 per cent. per annum. They thus form a sort of endowment, and if the money is not otherwise employed by the bondholder the society will redeem it at the subscriber's death, or when the bond has come to maturity. If, however, after paying the instalments due on his bond for three years he wants an advance on sufficient security, he can obtain one at the rate of £33 6s. 8d. for every 1s. 3d. subscription he has paid on the bond. Thus, if a bondholder has taken up a bond to run thirty years, and has paid on it 13s. 9d. a month for three years, he can get an advance of £350, for which he pays interest at the rate of 5 per cent. per annum. According to the intention for which the society is constituted, the advance would be granted for the purchase of a house, which would form the chief security of the society for the loan. If the rental of this house, which costs £350, is reckoned at £30 per annum, the bondholder can be shown to be making a good investment. His payments would be as follow:—

| | | | |
|---|---|---|---|
| Subscription on bond per annum ... ... | £8 | 5 | 0 |
| Interest on loan of £350 at 5 per cent. ... | 17 | 10 | 0 |
| Total payment per annum ... ... | £25 | 15 | 0 |

That is, £4 5s. less than we have estimated his rent to be. Moreover, at the maturity of the bond, the bondholder would receive £392 9s. 10d. After paying off the £350, the amount of his mortgage, he would have a surplus of £42 9s. 10d., and, in addition, he would own his house, whereas he might have been paying rent for the same time and not owned anything. As many people will make every effort to pay off a debt, though they would make little effort to save, this method of acquiring a house might be very useful.

The success of such a society as this must, however, depend very largely on the management.

